# Differential conformational dynamics in the closely homologous FK506-binding domains of FKBP51 and FKBP52

**DOI:** 10.1042/BJ20140232

**Published:** 2014-06-13

**Authors:** Sourajit M. Mustafi, David M. LeMaster, Griselda Hernández

**Affiliations:** *Wadsworth Center, New York State Department of Health, Empire State Plaza, Albany, NY 12201, U.S.A.; †Department of Biomedical Sciences, School of Public Health, University at Albany–SUNY, Empire State Plaza, Albany, NY 12201, U.S.A.

**Keywords:** conformational dynamics, differential line-broadening, FK506-binding protein of 51 kDa (FKBP51), FK506-binding protein of 52 kDa (FKBP52), mutational analysis, nuclear magnetic resonance (NMR), FKBP, FK506-binding protein, FRB, FKBP12–rapamycin-binding, Hsp, heat-shock protein, LBD, ligand-binding domain, NF-κB, nuclear factor κB, TPR, tetratricopeptide

## Abstract

As co-chaperones of Hsp90 (heat-shock protein 90), FKBP51 (FK506-binding protein of 51 kDa) and FKBP52 (FK506-binding protein of 52 kDa) act as antagonists in regulating the hormone affinity and nuclear transport of steroid receptor complexes. Exchange of Leu^119^ in FKBP51 for Pro^119^ in FKBP52 has been shown to largely reverse the steroid receptor activities of FKBP51 and FKBP52. To examine whether differences in conformational dynamics/plasticity might correlate with changes in the reported receptor activities, ^15^N-NMR relaxation measurements were carried out on the N-terminal FKBP domains of FKBP51 and FKBP52 as well as their residue-swapped variants. Both proteins exhibit a similar pattern of motion in the picosecond–nanosecond timeframe as well as a small degree of ^15^N line-broadening, indicative of motion in the microsecond–millisecond timeframe, in the β_3a_ strand of the central sheet. Only the FKBP51 domain exhibits much larger line-broadening in the adjacent β_3_ bulge (40′s loop of FKBP12) and throughout the long β_4_–β_5_ loop (80′s loop of FKBP12). The L119P mutation at the tip of the β_4_–β_5_ loop completely suppressed the line-broadening in this loop while partially suppressing the line-broadening in the neighbouring β_2_ and β_3a_ strands. The complementary P119L and P119L/P124S variants of FKBP52 yielded similar patterns of line-broadening for the β_4_–β_5_ loop as that for FKBP51, although only 20% and 60% as intense respectively. However, despite the close structural similarity in the packing interactions between the β_4_–β_5_ loop and the β_3a_ strand for FKBP51 and FKBP52, the line-broadening in the β_3a_ strand is unaffected by the P119L or P119L/P124S mutations in FKBP52.

## Abstract 2

Unlike FKBP52, the FK1 domain of FKBP51 exhibits microsecond–millisecond conformational dynamics in the β_3_ bulge and the β_4_–β_5_ loop, known sites of protein signalling interactions. Swapping residue 119 yields altered conformational dynamics in a pattern reminiscent of reported modulations in steroid receptor activity.

## INTRODUCTION

FKBP52 (FK506-binding protein of 52 kDa) is a high-molecular-mass member of the FKBP family that was first characterized as a co-chaperone of Hsp90 (heat-shock protein 90) in the activated progesterone, androgen and glucocorticoid receptor complexes [1]. The core complex of these steroid receptors, containing the steroid receptor protein, Hsp90 and p23, is formed by an ordered sequence of ATP-dependent protein-binding interactions that transiently involves Hsp40, Hsp70 and Hop (Hsp70/Hsp90-organizing protein) [2]. The mature complex is then established by the binding of a co-chaperone that bears a TPR (tetratricopeptide) repeat domain which interacts with Hsp90. This sophisticated assembly process stabilizes a receptor conformation that appears to optimize regulation of the signalling which results from steroid binding [2]. Although the conformational state of unliganded steroid receptors remains poorly characterized, crystal structures of various ligand-bound states demonstrate that the conformational transitions of the LBD (ligand-binding domain) that are induced by binding steroid antagonists generally differ from those induced by steroid agonists, and these distinct conformations can differentially interact with co-regulators [3]. Distinct LBD conformations have also been induced by the binding of non-steroidal glucocorticoid agonists, and in one such study, it has been argued that interaction with the non-steroidal agonist modulates a concurrent conformational transition in the Hsp90 component of the glucocorticoid receptor complex [4].

Although FKBP52 predominates in the steroid-bound receptor, the highly homologous FKBP51 is the predominant TPR co-chaperone in the unliganded state [5,6]. Steroid binding to the FKBP51-bound receptor is believed to induce the exchange for FKBP52 [7]. FKBP52 substitution increases the hormone-binding affinity of the receptor complex and enhances binding to the dynactin transport machinery, thereby facilitating transport of the receptor from the cytosol to the nucleus [8,9]. The resultant set of hormone-induced transcriptional activities includes an increased expression of FKBP51. Since the binding of FKBP51 lowers the hormone-binding affinity for the glucocorticoid and progesterone receptors, this increased production of FKBP51 provides a negative-feedback regulatory system. The activated androgen receptor also induces the transcription of the *FKBP5* gene (FKBP51). However, the relative activities of the FKBP51-bound and FKBP52-bound androgen receptors differ from that observed for the glucocorticoid and progesterone receptors such that a positive-feedback expression process can arise under pathological conditions which appears to often substantially contribute to the metastatic phase of prostate cancer [10–12].

Despite considerable effort, it has not yet been possible to reconstitute the effects of FKBP51 or FKBP52 in a biochemically defined reconstituted steroid hormone receptor system [2,13]. As a result, detailed insight into the biochemical and structural aspects of the steroid ligand-induced switching between FKBP51 and FKBP52 in the activated receptor complex remains problematic. Specific binding interactions between FKBP51/FKBP52 and the steroid receptor proteins have been proposed, including the BF-3 (binding function 3) regulatory site [14] and the H1–H3 loop [15] of the ligand-binding domain, although a direct binding interaction has not been demonstrated [16].

Using a yeast heterologous expression system for the human androgen receptor, Riggs et al. [17] demonstrated that a L119P mutation in the first FKBP domain (FK1) of human FKBP51 yielded a 3.5-fold increase in reporter gene expression. An additional A116V mutation doubled the potentiation to a level equivalent to that of the FKBP52-containing receptor complex. Introducing the complementary P119L mutation into FKBP52 yielded a smaller reverse effect (2-fold decreased reporter gene expression). Similar results were also obtained for these FKBP51 and FKBP52 variants in an embryonic fibroblast cell line derived from FKBP52-knockout mice [17].

Largely mediated via the FK1 domains, FKBP51 and FKBP52 also act as antagonists in regulating the phosphorylation state of the tau protein and its proper recycling [18,19]. In addition to its normal role in regulating microtubule polymerization, excessive tau phosphorylation contributes to the neurofibrillary tangles that are characteristic of various tauopathies. FKBP51 and FKBP52 are also believed to help to regulate the protein kinase Akt/PKB (protein kinase B) [20] and the transcription factor NF-κB (nuclear factor κB) [21–23]. Both Akt and NF-κB participate in the regulation of cell survival and apoptosis and are targets for major drug development programmes. Single nucleotide polymorphisms in the *FKBP5* gene strongly correlate with recurrence of depressive episodes, the rate of antidepressant response and psychological stress disorders [24,25]. Given the known clinical tolerance to extended inhibition across the FKBP domain protein family by non-immunosuppressant variants of FK506 [26,27], FKBP51 and FKBP52 are validated druggable proteins [13]. Considerable effort is currently directed towards developing selective inhibitors. Befitting the marked structural conservation between these two proteins [28–33], particularly in the catalytic active-site region, FK506-based drug design has so far failed to yield appreciable selectivity [30,31,34].

In the present study, ^15^N-NMR relaxation measurements were conducted on the FK1 domains of FKBP51 and FKBP52 as well as for variants at positions 119 and 124. The marked differences indicate substantial variations in the conformational sampling exhibited by FKBP51 and FKBP52 which may provide a basis for selective drug design as well as offer insight into localized conformational transitions that might contribute to the role of these two proteins in the ligand-induced activation of the steroid receptors.

## EXPERIMENTAL

### Protein preparation

Genes for the FK1 domains of human FKBP51 and FKBP52 as well as the variants at residues 119 and 124 were chemically synthesized (by GenScript) from the wild-type gene sequence, with codon optimization for expression in *Escherichia coli*. For FKBP52, a methionine start signal was introduced before Glu^21^ with the stop signal following Glu^140^. In the case of FKBP51, the construct began one residue earlier (Glu^20^), as inserting the methionine before Gln^21^ led to heterogeneous N-terminal processing. The genes were cloned into the expression vector pET11a and then transformed into the BL21(DE3) strain of *E. coli* (Novagen) for expression. The protein expression and purification procedure for both FKBP51 and FKBP52 followed that described previously for FKBP12 through the Sephadex G-50 size-exclusion chromatography step [35]. For FKBP52 samples, the eluent from the size-exclusion column was loaded on to a Q-Sepharose FF column equilibrated in 50 mM Tris/HCl, 30 mM acetic acid and 1 mM DTT (pH 8.0). The protein was then eluted with an NaCl gradient to 0.4 M in the same buffer solution. For FKBP51 samples, the eluent from the size-exclusion column was diluted 2-fold with water and loaded on to an SP-Sepharose FF column equilibrated in 25 mM Tris/HCl, 15 mM acetic acid and 1 mM DTT (pH 8.0). The protein was then eluted with an NaCl gradient to 0.4 M in the same buffer solution. All isotopically labelled samples were prepared via protein expression in minimal medium containing 0.1% ^15^NH_4_Cl as nitrogen source. For U-^13^C,^15^N-enriched samples, 0.2% [U-^13^C]glucose (Cambridge Isotopes) was substituted for the unlabelled glucose used for preparing the U-^15^N samples.

All protein samples were concentrated via centrifugal ultrafiltration (1 mM protein for FKBP52 and 0.5 mM protein for FKBP51) and then equilibrated into a pH 6.5 buffer containing 25 mM sodium phosphate, 2 mM DTT and 2 mM TCEP [tris(2-carboxyethyl)phosphine] by a series of centrifugal concentration steps.

### NMR spectroscopy

NMR assignment and relaxation data were collected on a Bruker Avance III 600 MHz spectrometer, a Bruker Avance II 700 MHz spectrometer, a Bruker Avance II 800 MHz spectrometer and a Bruker Avance II 900 MHz spectrometer at 25°C. Backbone resonance assignments (BMRB accession numbers 19787 and 19788 for FKBP51 and FKBP52 respectively) were carried out using standard HNCO [36], HN(CA)CO [36], HNCACB [37] and HN(CO)CACB [38] experiments. The proline *cis*–*trans* isomer analysis of FKBP52 was carried out using a 3D HCCCONH [39] experiment. HSQC-based T_1_, T_1ρ_ and heteronuclear NOE experiments were carried out as described by Lakomek et al. [40]. T_1_ relaxation delay periods of 0.08, 0.16 (×2), 0.24, 0.36, 0.48, 0.64 and 0.80 s were used at 600 MHz, whereas 0.08, 0.16 (×2), 0.24, 0.32, 0.40, 0.56 and 0.72 s were used at the higher fields with a recycle delay of 3 s. Again using a recycle delay of 3 s, the spin lock field was applied for periods of 10, 20, 30, 40, 60, 80, 100 and 120 ms at 600 and 800 MHz, whereas the last time period was removed for 900 MHz. Spin lock fields of 1245 Hz at 600 MHz, 1140 Hz at 800 MHz and 1085 Hz at 900 MHz were calibrated by offset-dependent scalar coupling measurements [41]. To enhance statistical sampling and minimize ^15^N offset corrections, the T_1ρ_ experiments were repeated at equally spaced ^15^N carrier frequencies (four sets at 600 and five sets at higher fields). For each resonance, the fitted exponentials for each set were then averaged according to the ^15^N offset using a linear weighting varying from 1.0 on resonance to 0.0 at an offset equal to 60% of the ^15^N spin lock field strength. A (10+1)-s saturation/recovery delay was used for duplicate runs of the heteronuclear NOE measurements [40]. Felix software (http://www.felixnmr.com) was used for NMR data processing and Modelfree 4.1 software [42] was used for the NMR relaxation analysis.

To maximize the sensitivity for detecting variations in the conformational exchange line-broadening effects between the wild-type proteins and their point mutants, the transverse relaxation rates were analysed in differential mode. For residues that do not exhibit conformational exchange line-broadening, the backbone ^15^N *R*_2_ values are generally dominated by the dynamics of global molecular tumbling which depends on sample viscosity. Since well fewer than half of the residues exhibit conformational exchange line-broadening, the median *R*_2_ values were used to normalize the relaxation effects arising from slightly differing sample temperatures and protein concentrations. The robustness of this normalization approach is indicated directly by the Δ*R*_2_ values for the residues not exhibiting significant differential line-broadening since these Δ*R*_2_ values incorporate the experimental errors of the individual relaxation measurements as well as the discrepancies that arise from a lack of equivalence for the conformational/orientational dynamics of the two protein samples. Since the relaxation effects of global molecular tumbling scale reasonably uniformly as a function of magnetic field, this differential *R*_2_ analysis could be usefully extended to comparing the 800 MHz and 900 MHz datasets obtained for FKBP52. Following median normalization of the FKBP52 variant datasets obtained at 800 MHz to the 900 MHz data for the wild-type protein, the Δ*R*_2_ values >1.0 s^−1^ were multiplied by the ratio of (9/8)^2^ to the median normalization factor. Variation by the square of the magnetic field corresponds to conformational line-broadening transitions that occur in the fast exchange regime as discussed below, whereas this magnetic field scaling approach takes advantage of the absence of conformational line-broadening in the β_4_–β_5_ loop of wild-type FKBP52.

## RESULTS AND DISCUSSION

### ^15^N relaxation measurements on the human FKBP52 FK1 domain

**Figure 1:**
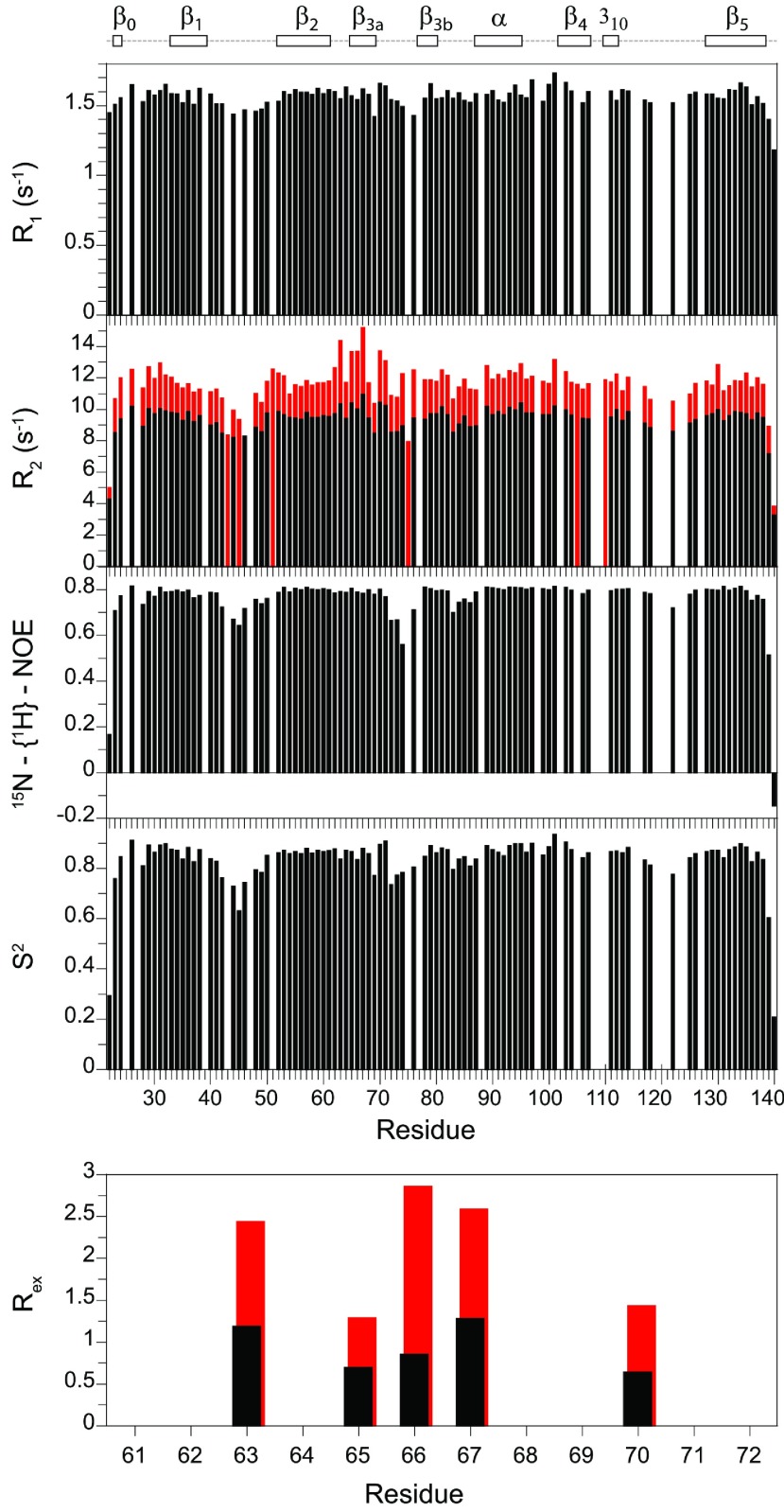
^15^N relaxation measurements for the backbone amide resonances in the FK1 domain of FKBP52 at 25°C The longitudinal (*R*_1_) and transverse (*R*_2_) relaxation rates at 600 MHz ^1^H are shown with the transverse relaxation rates at 900 MHz ^1^H also indicated in red. The heteronuclear NOE and model-free [48,49] order parameters (*S*^2^) are also illustrated along with the conformational exchange line-broadening *R*_ex_ values for residues in the β_3a_ strand. In addition to proline residues, relaxation data are not reported for overlapped resonances and for the severely broadened resonance of Ser^115^.

**Figure 2:**
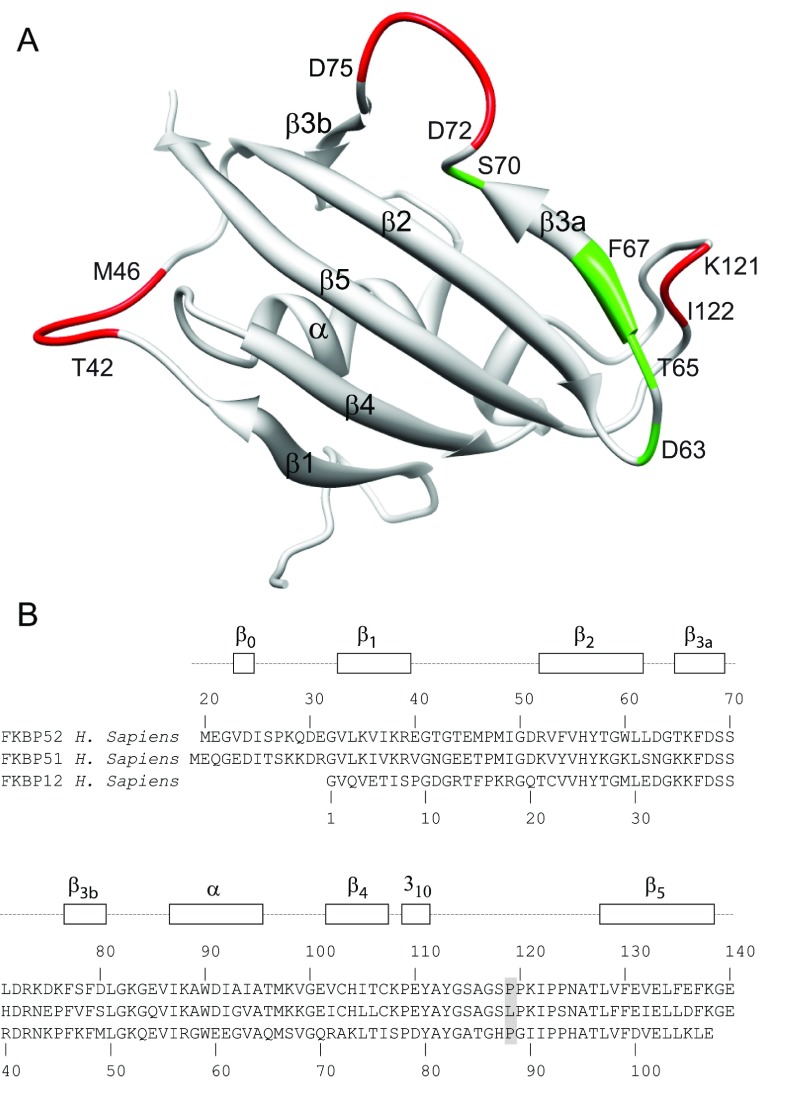
Structural distribution for residues of the FK1 domain of FKBP52 that exhibit conformational dynamics in either the picosecond–nanosecond or microsecond–millisecond timeframe (**A**) Main-chain conformational schematic diagram of the FK1 domain as viewed from the back side of the β-sheet. Discounting the termini, residues that exhibit order parameter values of *S*^2^<0.78 are indicated in red, whereas those exhibiting conformational exchange broadening above 0.5 s^−1^ at 600 MHz ^1^H and above 1.0 s^−1^ at 900 MHz ^1^H are indicated in green. (**B**) Sequence alignment of the FK1 domains of FKBP52 and FKBP51 with FKBP12. Residue 119 at the tip of the β_4_–β_5_ loop is highlighted.

On the basis of their earlier assignment of ^1^H and ^15^N resonances for the N-terminal domain of rabbit FKBP52 [43], Craescu et al. [44] obtained the first structural determination of this domain which demonstrated a close structural homology with FKBP12 with an additional short β-strand at the beginning of the domain (herein denoted β_0_). We carried out backbone resonance assignment on a U-^13^C,^15^N-labelled sample of the human FK1 domain (Glu^21^–Glu^140^) which differed only modestly from the earlier analysis [43] due to the four residue substitutions between the two species. ^13^C assignments for various side-chain positions were also obtained as discussed below. Longitudinal (*R*_1_) and transverse (*R*_2_) ^15^N relaxation rates as well as ^15^N heteronuclear NOE values were determined (Figure 1). In three segments of the sequence, all three relaxation values are significantly decreased, indicative of increased internal motion in the picosecond–nanosecond timeframe. No comparable decrease in relaxation values was observed for any segment throughout the backbone of FKBP12 [35]. The first such segment with enhanced fast internal motion (Thr^42^–Met^46^) corresponds to the outer strand of a topological crossing of two loops in FKBP52 that connect β-strands of the central sheet (Figure 2). As noted in the early crystal structures of FKBP12 [45], such a topology is rarely observed in antiparallel β-sheets [46]. In the structurally related FKBP13, this loop crossing is stabilized by a disulfide bridge linking the two loops [47]. Decreased relaxation values were also observed for Asp^72^–Asp^75^ which form a bulge in the centre of the β_3_ strand (referred to as the 40′s loop in FKBP12). Finally, there also appears to be enhanced picosecond–nanosecond motion near the tip of the long β_4_–β_5_ loop (the 80′s loop of FKBP12) although the ^15^N relaxation data are quite sparse, primarily due to the presence of four proline residues at positions 119, 120, 123 and 124.

Elevated *R*_2_ values, particularly when that effect is enhanced as a function of magnetic field strength, generally arise from conformational transitions that occur in the microsecond–millisecond timescale. Only one region of the FK1 domain of FKBP52 exhibits a small amount of the resonance line-broadening from this timescale of motion. The residues most affected lie within the β_3a_ strand and extend up to the start of the β-bulge (Figure 2). Such line-broadening transitions within the strands of a β-sheet are relatively uncommon. It is reasonable to suspect that the microsecond–millisecond motion giving rise to this modestly elevated line-broadening extends into the β-bulge segment as well, but the relaxation effects of that motion are obscured by the picosecond–nanosecond motions that yielded reduced *R*_2_ and *R*_1_ relaxation values. When significant mobility in both of these time regimes is present, the standard three relaxation experiments generally provide insufficient constraints to adequately characterize the dynamics.

### *Cis*–*trans* isomerization state of Pro^120^ in the FK1 domain of FKBP52

When model-free analysis [48,49] was applied to these FKBP52 relaxation data, it was apparent that there is a small degree of anisotropy in the rotational diffusion of the protein (*D*_||_/*D*_⊥_ of 1.12). Further analysis requires the specification of a structural model. A significant issue regarding the conformation of the FKBP52 FK1 domain in solution is the configuration of the Pro^120^ linkage at the tip of the β_4_–β_5_ loop. Among the four crystal forms solved to date, two (PDB codes 1N1A [50] and 4LAV [33]) have a *cis*-proline residue, whereas the other two (PDB codes 1Q1C [51] and 4LAW [33]) have a *trans*-proline peptide bond.

The C^β^ and C^γ^ chemical shifts of proline residues depend upon the equilibrium of the ring pucker distribution which, in turn, depends upon the *cis*–*trans* equilibrium of the peptide linkage [52]. For *trans*-proline residues, the differences between the C^β^ and C^γ^ chemical shifts are close to 4.5 p.p.m., whereas corresponding values for *cis*-proline residues are near 9.6 p.p.m. [53]. A 2D CT (constant time)-HSQC experiment resolved the ^1^H^δ^-^13^C^δ^ resonances for all seven proline residues. In contrast with the more rapid conformational line-broadening transitions, *cis*–*trans* peptide transitions generally occur in the timeframe of seconds or longer so that distinct NMR resonances are observed for *cis* and *trans* states. With the proline ^1^H^δ^-^13^C^δ^ resonances of FKBP52 exhibiting an average S/N ratio above 50, no evidence of peak doublings arising from prolyl isomerization was detected. The large 10.0 p.p.m. difference in C^β^ and C^γ^ chemical shifts for Pro^120^ indicates a *cis*-peptide linkage for that residue (Supplementary Figure S1 at http://www.biochemj.org/bj/461/bj4610115add.htm).

### Backbone resonance assignment and ^15^N relaxation analysis of the FKBP51 FK1 domain

The human FKBP51 protein sequence from Glu^20^ to Glu^140^ was expressed in *E. coli* with U-^13^C,^15^N labelling. Backbone and ^13^C^β^ assignments were obtained for all resonances, except for the amide resonances of Ser^115^, which is presumably broadened beyond detection due to rapid hydrogen exchange as observed for the homologous position in FKBP12 [54] and FKBP52 [43]. The amide resonances in the 2D ^1^H-^15^N-HSQC spectrum of the FKBP51 FK1 domain are well dispersed and appear readily amenable to relaxation analysis (Supplementary Figure S2 at http://www.biochemj.org/bj/461/bj4610115add.htm). However, preliminary relaxation measurements on a 1 mM sample yielded *R*_1_ and *R*_2_ values that were appreciably larger than those for the similar sized FKBP52 FK1 domain, consistent with weak aggregation. Dilution of the FKBP51 FK1 domain to 0.5 mM yielded a global correlation time from ^15^N relaxation analysis which was only 5% larger than that observed for the FKBP52 domain while still providing a satisfactory level of signal intensity.

**Figure 3:**
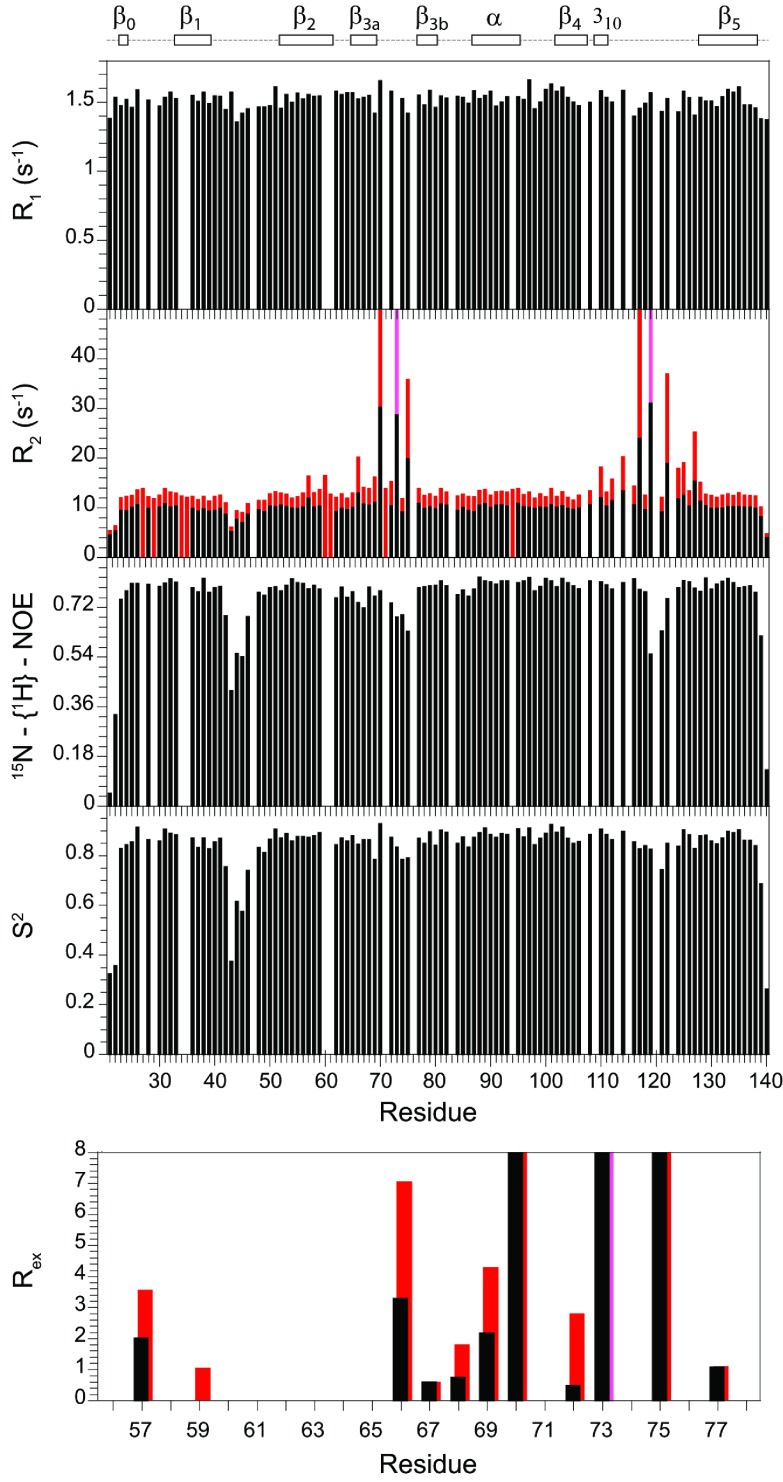
^15^N relaxation measurements for the backbone amide resonances in the FK1 domain of FKBP51 at 25°C The longitudinal (*R*_1_) and transverse (*R*_2_) relaxation rates at 600 MHz ^1^H are shown with the transverse relaxation rates at 900 MHz ^1^H also indicated in red. The *R*_2_ values at 900 MHz for Arg^73^ and Leu^119^ are illustrated in pink, indicating rates that are significantly above 50 s^−1^ for which the attenuated resonances could not be reliably quantified. The heteronuclear NOE and model-free [48,49] order parameters (*S*^2^) are also illustrated along with the conformational exchange line-broadening *R*_ex_ values for residues extending from within the β_2_ strand to the end of the β_3_ bulge. *R*_ex_ values for Ser^70^, Arg^73^ and Glu^75^ are truncated to better illustrate the smaller line-broadening effects in the β_2_ and β_3a_ strands. In addition to proline residues, relaxation data are not reported for overlapped resonances and for the severely broadened resonances of Tyr^113^ and Ser^115^.

**Figure 4:**
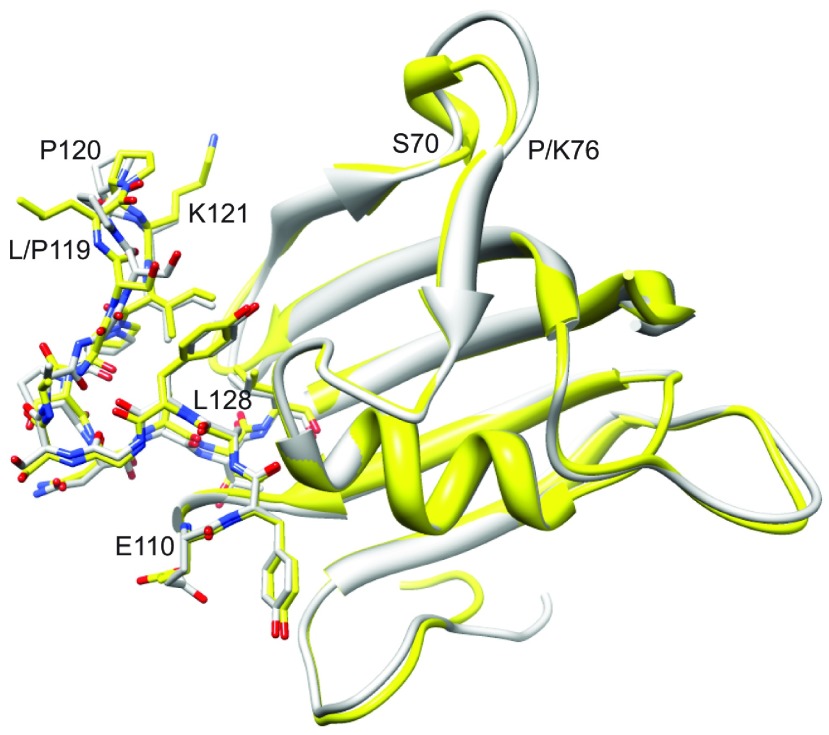
Superimposition of the FK1 domains of FKBP51 and FKBP52 The FKBP51 X-ray structure from PDB code 3O5P [28] is illustrated in yellow, whereas molecule A from PDB code 4LAV [33] for FKBP52 is shown in grey. All heavy atoms are illustrated for the β_4_–β_5_ loop extending from Glu^110^ to Leu^128^. Substantial deviations in backbone geometry are only apparent for the β_3_ bulge (Ser^70^–Lys^76^) and the tip of the β_4_–β_5_ loop.

The ^15^N relaxation data for the FK1 domain of FKBP51 (Figure 3) markedly differ from that observed for the FKBP52 domain (Figure 1). Strongly elevated *R*_2_ values are observed for residues in the β_3_ bulge and for many of the residues throughout the long β_4_–β_5_ loop (Figure 4). These elevated *R*_2_ values were closely similar for the 0.5 mM and 1 mM samples, indicating that they did not arise from the dynamics of weak aggregation interactions. The magnetic-field-dependence of these elevated *R*_2_ values indicates substantial line-broadening arising from motion in the sub-millisecond timeframe. The three residues of the β_3_ bulge exhibiting the largest line-broadening effects (Ser^70^, Arg^73^ and Glu^75^) have *R*_2_ values that are closely similar to those observed in FKBP12 [35]. On the other hand, additional smaller conformational line-broadening effects are observed for residues within the β_3a_ strand (Figure 3) as well as cross-strand interactions with the amides of Tyr^57^ and Gly^59^ in the β_2_ strand which hydrogen-bond in the X-ray structure with the side-chain O^γ^ of Ser^70^ and the carbonyl oxygen of Asp^68^ respectively. In analysing the magnetic-field-dependence of the ^15^N *R*_2_ relaxation values for the residues of the β_4_–β_5_ loop (Supplementary Figure S3 at http://www.biochemj.org/bj/461/bj4610115add.htm) as well as for those in the β_3a_ strand and β_3_ bulge, the increase in conformational line-broadening is approximately proportional to the square of the magnetic field. This implies that the conformational transition rate(s) is substantially higher than the strength of the spinlock field used in the *R*_1ρ_ experiments (1245 Hz at 600 MHz and 1085 Hz at 900 MHz), approaching the fast exchange limit as reported previously for FKBP12 [55–57]. The magnitude of conformational line-broadening depends upon the relative population and rate of interchange between the conformer states as well as on the differential ^15^N chemical shifts for these states. Near the fast exchange limit, the relative magnitude of these three contributions cannot be reliably deconvoluted.

Although FKBP12 also exhibits line-broadening conformational dynamics in the β_4_–β_5_ loop, that transition appears to be centred around the flip from a positive to a negative main-chain ϕ torsion angle at Gly^89^ [58]. The analogous residue in FKBP51 and FKBP52 is Pro^120^ which is covalently constrained from adopting a positive ϕ torsion angle. This implies that the line-broadening conformational transition observed in FKBP51 differs mechanistically from the transition reported for FKBP12. With respect to motions in this microsecond–millisecond timeframe, the FK1 domains of FKBP51 and FKBP52 are strikingly different despite a high degree of sequence homology. In particular, for the sequence from Cys^107^ to Leu^128^ encompassing the long β_4_–β_5_ loop, the two proteins differ at only residues 119 and 124. As discussed above, introduction of the L119P mutation into FKBP51 is sufficient to induce androgen receptor activation up to nearly the level observed for FKBP52 [17]. Conversely, introduction of the P119L mutation into FKBP52 partially reduces the level of steroid-induced receptor activation. To determine the degree to which these mutations might similarly alter the conformational transitions of FKBP51 and FKBP52, relaxation analysis of mutational variants at residues 119 and 124 was conducted.

### Dynamical effects of the L119P substitution in the FKBP51 FK1 domain

**Figure 5:**
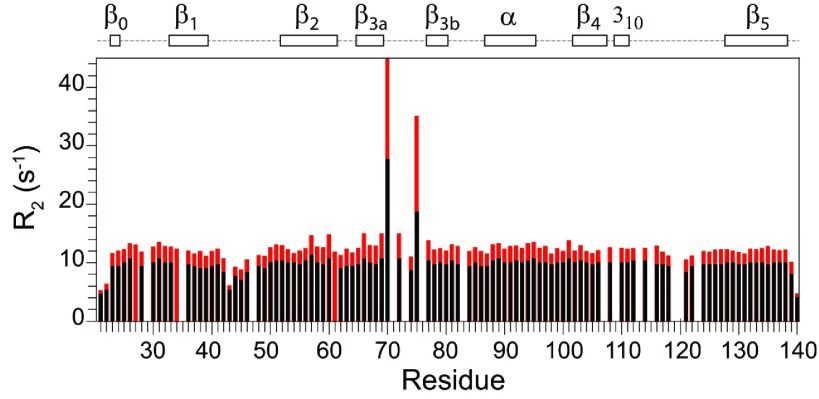
^15^N transverse relaxation measurements for the L119P variant of FKBP51 at 25°C The transverse (*R*_2_) relaxation rates at 600 MHz ^1^H are shown with the transverse relaxation rates at 900 MHz ^1^H also indicated in red.

**Figure 6:**
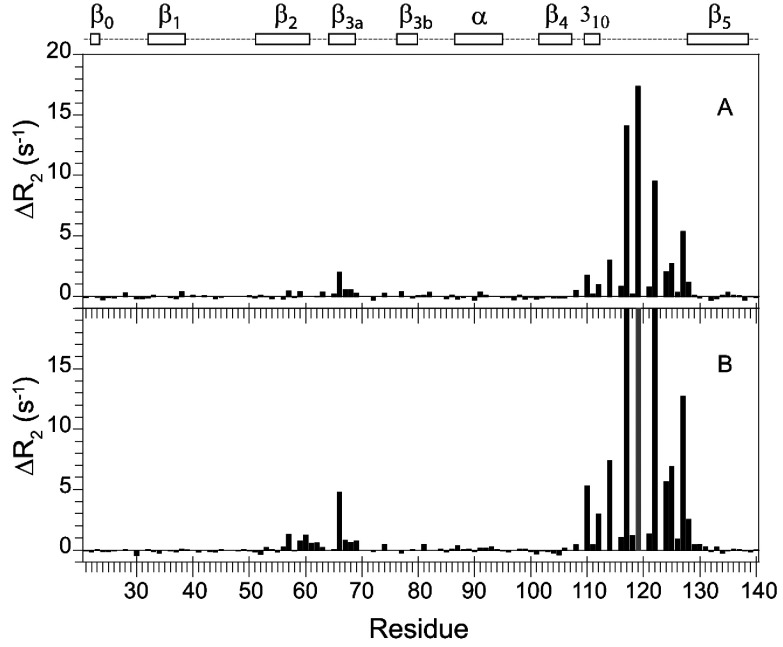
Differential ^15^N transverse relaxation measurements for the wild-type and L119P variant of FKBP51 at 25°C The differential transverse relaxation rates at 600 MHz ^1^H are shown in (**A**), whereas those for 900 MHz ^1^H are shown in (**B**). The data for the two fields are plotted on the same vertical scale to illustrate the approximate 2.25-fold increase for the 900 MHz data indicative of conformational transitions occurring near the fast exchange time limit. As a result, the Δ*R*_2_ values for residues 117, 119 and 122 at 900 MHz are truncated. At each field, the median *R*_2_ values for the two datasets are scaled to correct for small variations in the global molecular correlation times. Outside the regions exhibiting significant differential line-broadening (i.e. residues 57–77 and 108–128), the RMSD for the Δ*R*_2_ values were 0.15 and 0.18 s^−1^ for 600 MHz and 900 MHz respectively, corresponding to 1.5% of the median *R*_2_ values in each case. The Δ*R*_2_ value for Leu^119^ in the wild-type protein is given relative to the median *R*_2_ value and at 900 MHz this Δ*R*_2_ value is too large for reliable quantification (grey). Owing to decreased statistical reliability for the more severely attenuated resonances, the residues in which the *R*_2_ value is >18 s^−1^ for both wild-type and the L119P variant were excluded (Ser^70^, Arg^73^ and Glu^75^).

**Figure 7:**
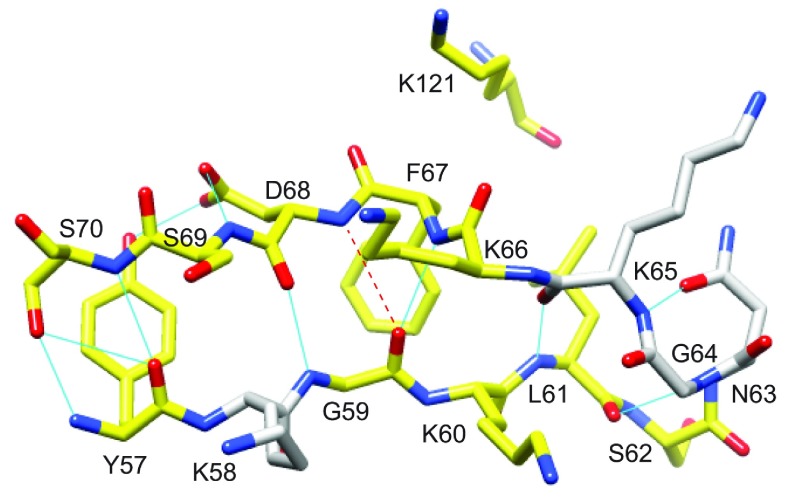
Structural distribution of residues in the β_2_ and β_3a_ strands of FKBP51 that exhibit reductions in *R*_2_ values resulting from the L119P substitution Residues for which the ^15^N *R*_2_ value decreases by more than 0.5 s^−1^ at 900 MHz ^1^H are coloured yellow. There are no other differences in *R*_2_ greater than 0.5 s^−1^ outside the β_4_–β_5_ loop. A kink in the β_3a_ strand occurs at Phe^67^ and Asp^68^ where the amide hydrogen of Asp^68^ is slightly too far from the carbonyl oxygen of Gly^59^ to form a canonical antiparallel β-sheet hydrogen-bonding interaction. This kink occurs at the site of direct contact with the tip of the β_4_–β_5_ loop as indicated by Lys^121^.

The 2D ^1^H,^15^N-HSQC spectrum for the FK1 domain of FKBP51 is largely unperturbed when the L119P mutation was introduced (Supplementary Figure S2). Despite this evidence for similarity in tertiary structure, the conformational exchange line-broadening in the β_4_–β_5_ loop was completely suppressed in the L119P variant (Figure 5). The pattern of line-broadening in the β_3a_ strand and β_3_ bulge is quite similar to that observed for the wild-type protein, although the magnitude of that line-broadening appears to be somewhat diminished. To analyse the line-broadening in the wild-type and L119P variant FK1 domain more directly, the differences in these *R*_2_ values were considered for both the 600 MHz and 900 MHz datasets (Figure 6). In addition to the large changes in conformational exchange line-broadening in the β_4_–β_5_ loop, introduction of the L119P mutation also partially suppresses the line-broadening in the β_2_ and β_3a_ strands. The largest effects in these strands occur near the highly conserved Leu^61^ and Phe^67^ whose side chains pack tightly against the similarly highly conserved Ile^122^ and Pro^123^ side chains at the tip of the β_4_–β_5_ loop. These L119P-induced changes in conformational exchange line-broadening extend along the β_2_ and β_3a_ strands and possibly into the β_3_ bulge (Figure 7). As a potential component of the conformational transition that underlies this line-broadening behaviour, it may be noted that the canonical antiparallel hydrogen-bonding pattern between the β_2_ and β_3a_ strands is disrupted by the amides of both Phe^67^ and Asp^68^ being oriented towards the carbonyl oxygen of Gly^59^ with the Asp^68^ amide being just beyond hydrogen-bonding distance (Figure 7).

It is interesting to consider this previously uncharacterized dynamical cross-talk between the β_4_–β_5_ loop and the β_2_ and β_3a_ strands and β_3_ bulge with respect to the recently reported interactions of the FK1 domain of FKBP51 in a rapamycin-mediated complex with the FRB domain of mTOR (mammalian target of rapamycin). Hausch and colleagues have reported that, whereas both the β_4_–β_5_ loop and the β_3_ bulge interact directly with the FRB (FKBP12–rapamycin-binding) domain, the β_3_ bulge contributes a larger proportion of these interactions [32]. The geometry of the FKBP51-bound ternary complex differs significantly from that reported previously for the ternary complex of FKBP12 with rapamycin and the FRB domain in which the β_4_–β_5_ loop of FKBP12 provides the predominant set of interprotein interactions [59].

### Dynamic effects of the P119L and P124S substitutions in the FKBP52 FK1 domain

**Figure 8:**
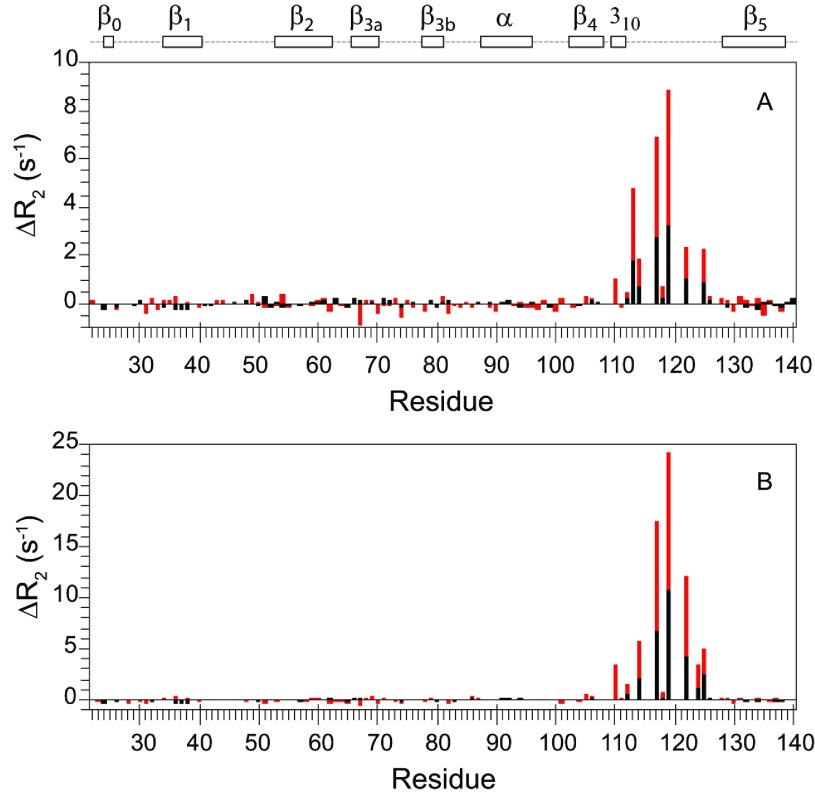
Differential ^15^N transverse relaxation measurements for the P119L and P119L/P124S variants of FKBP52 compared with the wild-type protein Relative to wild-type FKBP52, the differential transverse relaxation rates at 600 MHz (black) and higher field (red) are shown for P119L (**A**) and P119L/P124S (**B**). At each field, the median *R*_2_ values for the two datasets are scaled to correct for small variations in the global molecular correlation times. The higher field data for the two variants of FKBP52, collected at 800 MHz, were scaled to the wild-type FKBP52 data at 900 MHz under the assumption that the conformational exchange occurs in the fast limit regime. Outside the region exhibiting significant differential line-broadening (i.e. residues 110–125), the RMSD for the Δ*R*_2_ values were 0.13 and 0.18 s^−1^ for 600 MHz and higher field data for the P119L/P124S variant comparison respectively. The Δ*R*_2_ values for Leu^119^ and Ser^124^ are given relative to the median *R*_2_ value.

As discussed above, the β_4_–β_5_ loop in the FK1 domain of FKBP52 exhibits no evidence of conformational exchange line-broadening, indicative of motion in the microsecond–millisecond timeframe. Introduction of the P119L mutation into FKBP52 induces line-broadening dynamics in this loop (Figure 8A), although comparison of the 2D ^1^H,^15^N-HSQC spectra indicated minimal change in structure (Supplementary Figure S4 at http://www.biochemj.org/bj/461/bj4610115add.htm). The distribution of the increased *R*_2_ relaxation rates among the residues in the β_4_–β_5_ loop is qualitatively similar to that observed for the wild-type FK1 domain of FKBP51, although the magnitude of the effects are ~5-fold smaller (Supplementary Figure S3). In comparing with the L119P mutation-induced line-broadening effects for the β_2_ and β_3a_ strands of FKBP51, no similar enhancement in *R*_2_ values was apparent for these strands in FKBP52 following the P119L substitution (Figure 8A).

The fact that the pattern of altered *R*_2_ relaxation rates among the residues in the β_4_–β_5_ loop is qualitatively similar upon substituting L119P into FKBP51 compared with substituting P119L into FKBP52 strongly suggests that a similar conformational transition is being affected in both systems. On the other hand, the magnitude of the line-broadening effects is substantially smaller for the P119L substitution into FKBP52 than that observed when the L119P mutation is introduced into FKBP51, reflecting either differences in the conformer populations or in the rates of interchange. With regard to the possibility that these variations in conformational dynamics might reflect alterations in biological functionality, as noted above, Riggs et al. [17] observed a similar pattern of a smaller proportional decrease in transcriptional expression upon substituting P119L into FKBP52 compared with the larger proportional increase in expression that they observed upon substituting L119P into FKBP51.

Given that the β_4_–β_5_ loops of FKBP51 and FKBP52 differ only at residues 119 and 124, we examined the relaxation behaviour of the FKBP51-like P119L/P124S double mutant of FKBP52. Riggs et al. [17] reported that the P124S mutation had minimal effects upon the observed transcription levels of FKBP52 whether introduced into wild-type FKBP52 or into the P119L background. We observed that the differential line-broadening within the β_4_–β_5_ loop increased 3-fold for the P119L/P124S double mutant of FKBP52, relative to the P119L variant, yielding a line-broadening effect that was 60% of that observed for FKBP51 (Supplementary Figure S3). Once again, the pattern of differential line-broadening along the residues of the β_4_–β_5_ loop was quite similar to that for the P119L variant of FKBP52 and to that for FKBP51, suggesting that a similar conformational transition is being monitored.

As with the P119L variant, the differential line-broadening induced by the double mutation of FKBP52 does not propagate from the β_4_–β_5_ loop into the β_3a_ strand (Figure 8B) as it does for FKBP51 (Figure 6). The structural basis for this differential propagation of conformational dynamics is not apparent from examination of the crystal structures of the wild-type proteins. Residues 119 and 124 do not contact the β_2_ or β_3a_ strands directly and they do not appear to significantly distort the conformation of residues 121–123 that form those contacts. The residues of those strands on that interface are evolutionarily conserved and are positioned quite similarly in the crystal structures of FKBP51 and FKBP52.

Despite their substantial structural similarity, in both the β_3_ bulge and the β_4_–β_5_ loop, the FK1 domain of FKBP51 undergoes significantly populated conformational transitions that appear to be suppressed in FKBP52. Given the difficulties reported to date in developing lead compounds to discriminate between these two proteins, these transiently sampled conformations of FKBP51 might provide useful targets for further design. As the changes in ^15^N chemical shifts that underlie the resonance line-broadening process are primarily dependent on the backbone torsion angles [60], the substantially elevated *R*_2_ values from Glu^110^ to Leu^128^ across the entire β_4_–β_5_ loop as well as into the β_3a_ strand suggest an appreciable alteration of structure in this region of the protein. The correspondence between the mutationally induced changes in conformational dynamics of the β_4_–β_5_ loop monitored by NMR relaxation and the alterations in the transcriptional activity of the androgen receptor complexes containing these various FKBP51 and FKBP52 sequences warrants further consideration. The ligand-induced conformational transitions in the ligand-binding domain of the steroid receptors have long garnered intense research interest, although as yet providing an incomplete structural understanding. In the effort to analyse this system further, a concurrent conformational transition in Hsp90 has been proposed [4]. It may likewise prove useful to consider whether the conformational transition in the β_4_–β_5_ loop of FKBP51 monitored in the present study might play a role in the initial binding of FKBP51 to the unliganded steroid receptor and/or in the steroid-induced release of FKBP51 from the complex.

## Online data

Supplementary data
