# Supplementary material for: Differential conformational dynamics in the closely homologous FK506-binding domains of FKBP51 and FKBP52
Source: Biochem J. 2014 Jun 13;461(Pt 1):115–23. doi: 10.1042/BJ20140232 (PMC4060953; doi:10.1042/BJ20140232)
Supplement: Supplementary data [file bj4610115add.pdf]

Biochem. J. (2014) 461, 115–123 (Printed in Great Britain) doi:10.1042/BJ20140232

SUPPLEMENTARY ONLINE DATA

Differential conformational dynamics in the closely homologous FK506-binding domains of FKBP51 and FKBP52

Sourajit M. MUSTAFI*, David M. LeMASTER*† and Griselda HERNÁNDEZ*†[1]

*Wadsworth Center, New York State Department of Health, Empire State Plaza, Albany, NY 12201, U.S.A.
†Department of Biomedical Sciences, School of Public Health, University at Albany – SUNY, Empire State Plaza, Albany, NY 12201, U.S.A.

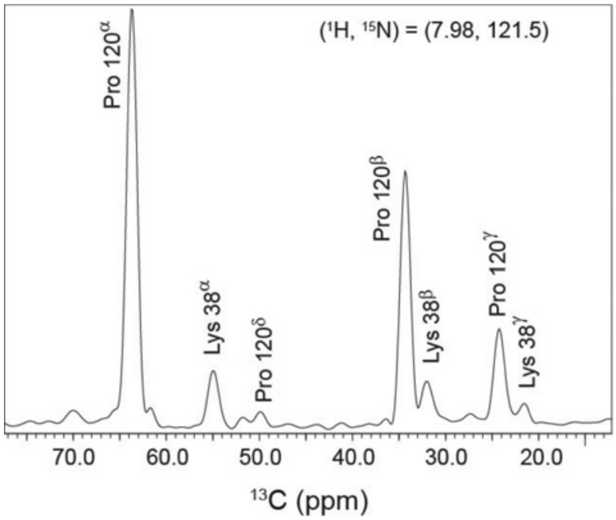

Figure S1 1D slice from a 3D HCCCONH NMR experiment on the U-^{13}C,^{15}N-enriched FK1 domain of FKBP52

Correlations to the amide ^{1}H and ^{15}N frequencies for Lys^{121} were observed for the side-chain ^{13}C resonances of Pro^{120}, indicating a 10.0 p.p.m. separation for the C$^\beta$ and C$^\gamma$ resonances. Partial overlap was also observed for the ^{13}C correlations of Lys^{38} to the nearby ^{1}H and ^{15}N frequencies for the amide of Arg^{39}.

[1] To whom correspondence should be addressed (email gch02@health.state.ny.us).
Backbone resonance assignments for FKBP51 and FKBP52 have been deposited in the BMRB under accession numbers 19787 and 19788 respectively.

© 2014 The Author(s)
The author(s) has paid for this article to be freely available under the terms of the Creative Commons Attribution Licence (CC-BY) (http://creativecommons.org/licenses/by/3.0/) which permits unrestricted use, distribution and reproduction in any medium, provided the original work is properly cited.

Biochemical Journal www.biochemj.org

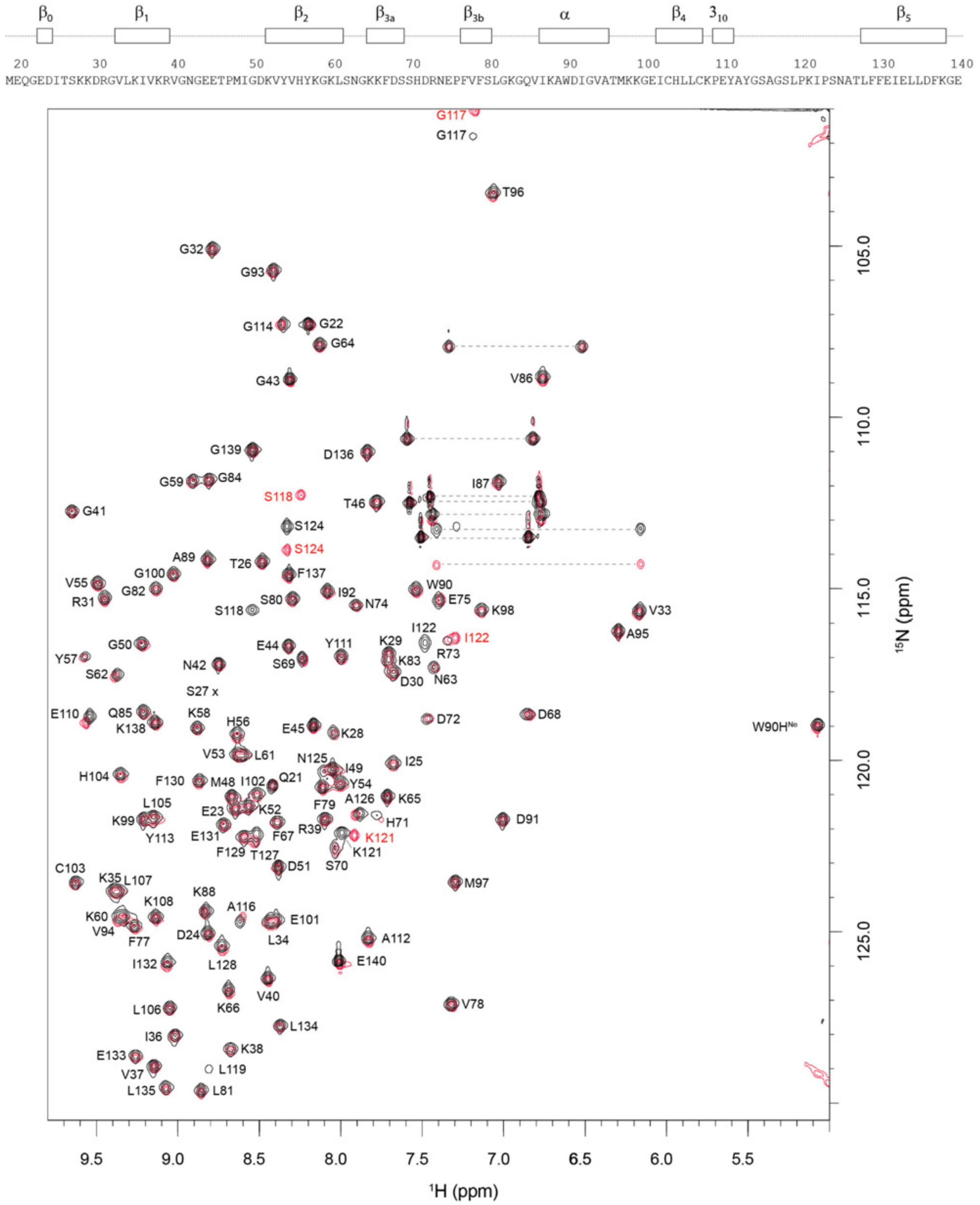

Figure S2 2D ^{1}H-^{15}N-NMR correlation spectra for ^{15}N enriched the wild-type (black) and the L119P variant (red) of FKBP51

Resonances for side-chain amides are connected by broken lines.

© 2014 The Author(s)
The author(s) has paid for this article to be freely available under the terms of the Creative Commons Attribution Licence (CC-BY) (http://creativecommons.org/licenses/by/3.0/) which permits unrestricted use, distribution and reproduction in any medium, provided the original work is properly cited.

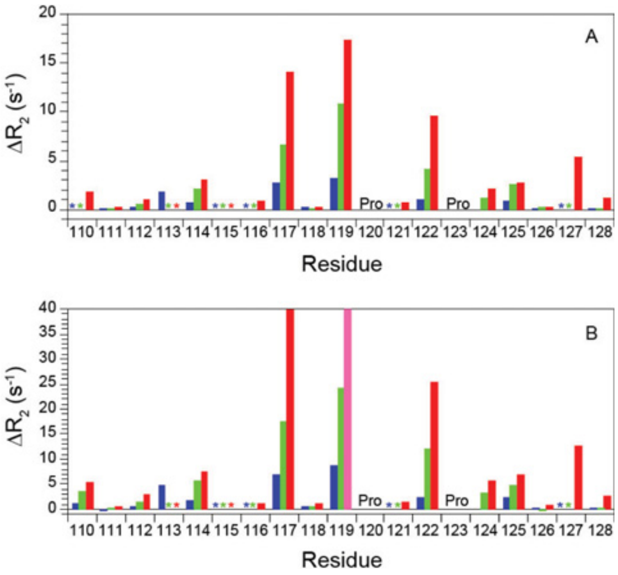

Figure S3 Differential transverse relaxation rates in the β_4–β_5 loop for the P119L (blue) and P119L/P124S (green) variants of FKBP52 and the L119P variant (red) of FKBP51 compared with the wild-type proteins at 600 MHz (A) and higher field (B)

Asterisks denote residues for which well-resolved decay profiles were not obtained. For residue 119 of FKBP51, the attenuated peak intensities at 900 MHz precluded a reliable rate determination, although the value is $>50\ s^{-1}$ (pink). The higher field data for the two variants of FKBP52 were collected at 800 MHz, whereas 900 MHz was used for the other samples. Following normalization to the mean R_2 value for the wild-type FKBP52 data, for residues exhibiting a differential larger than $1.0\ s^{-1}$ that difference was scaled by the square of the magnetic field $(9/8)^2$, consistent with motion near the fast exchange limit.

© 2014 The Author(s)
The author(s) has paid for this article to be freely available under the terms of the Creative Commons Attribution Licence (CC-BY) (http://creativecommons.org/licenses/by/3.0/) which permits unrestricted use, distribution and reproduction in any medium, provided the original work is properly cited.

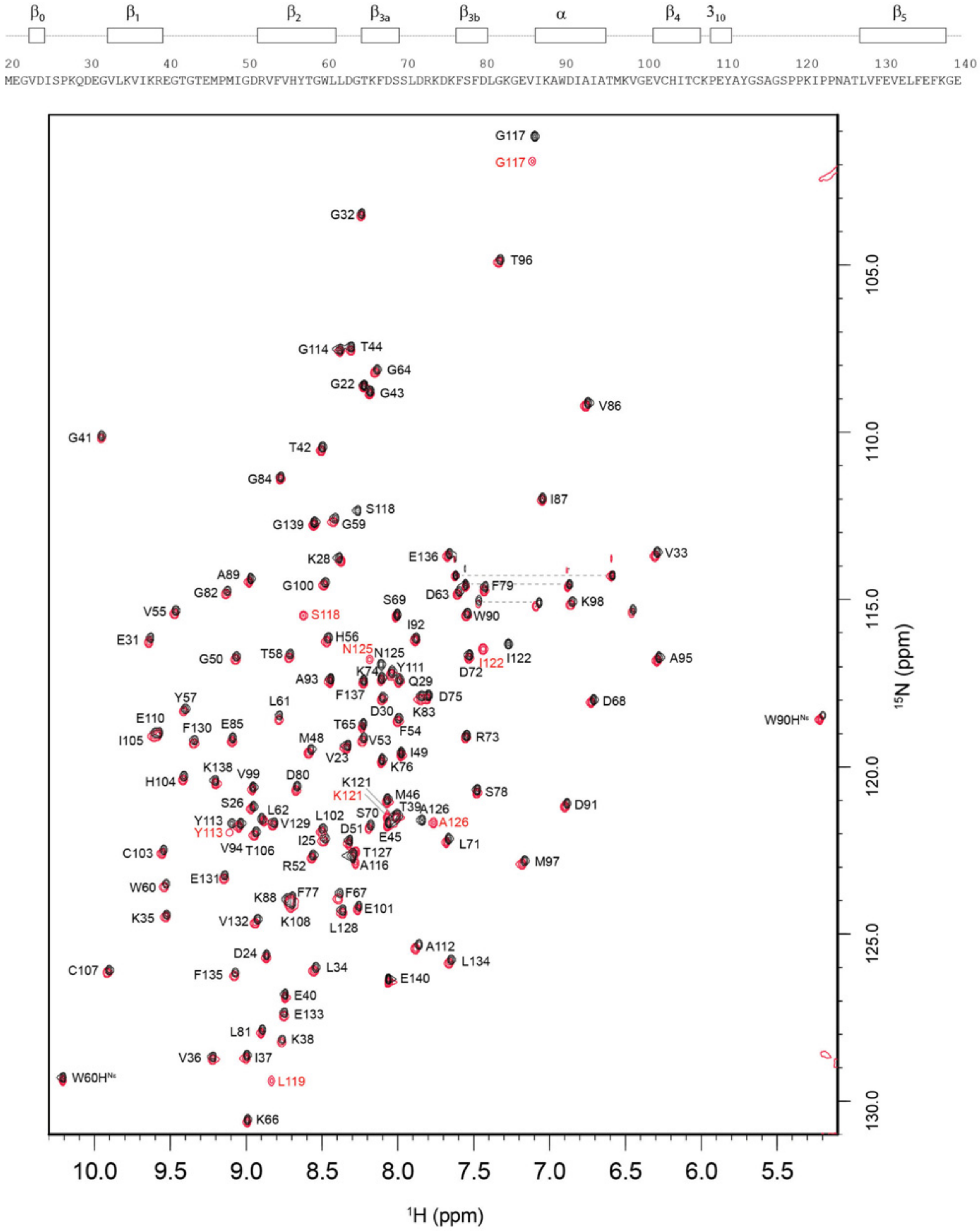

Figure S4 2D ^{1}H-^{15}N-NMR correlation spectra for ^{15}N enriched the wild-type (black) and the P119L variant (red) of FKBP52

Resonances for side-chain amides are connected by broken lines.

Received 19 February 2014/27 March 2014; accepted 22 April 2014
Published as BJ Immediate Publication 22 April 2014, doi:10.1042/BJ20140232

© 2014 The Author(s)
The author(s) has paid for this article to be freely available under the terms of the Creative Commons Attribution Licence (CC-BY) (http://creativecommons.org/licenses/by/3.0/) which permits unrestricted use, distribution and reproduction in any medium, provided the original work is properly cited.
